# Genomics-enabled dissection of sea wheatgrass genome for advancing wheat genetic resources

**DOI:** 10.1007/s00122-025-05021-8

**Published:** 2025-09-18

**Authors:** Dilkaran Singh, Qijun Zhang, Ghana Challa, Elias M. Elias, Steven S. Xu, Wanlong Li

**Affiliations:** 1 Department of Biology and Microbiology, South Dakota State University, Brookings, SD 57007 USA; 2 Department of Plant Science, North Dakota State University, Fargo, ND 58108 USA; 3 USDA-ARS, Western Regional Research Center, Albany, CA 94710 USA; 4 Present Address: Department of Plant Biology, University of Illinois, 1201 W. Gregory Dr., Urbana, IL 61801 USA; 5 Present Address: Altius Institute for Biomedical Sciences, 2211 Elliot Ave, Seattle, WA 98121 USA

## Abstract

Wheat production is challenged by biotic and abiotic stresses. Alien gene transfer is an effective approach to tackle such challenges. We previously showed that sea wheatgrass (SWG; *Thinopyrum junceiforme* (2*n* = 2*x* = 28; J_1_J_2_) is an untapped resource possessing resistance to an array of pests and abiotic stress. However, the transfer of these important traits has been hindered by the lack of genomic resources and a clear picture of its genome constitution. Using multi-color genomic in situ hybridization, we distinguished the SWG sub-genomes and corroborated that the J_1_ sub-genome is closely related to the E genome of *Th. elongatum* and the J genome of *Th. bessarabicum* and the J_2_ sub-genome to the V genome of *Dasypyrum villosum*. Meanwhile, we developed a draft SWG genome assembly and 127 SWG-specific DNA markers covering the 14 SWG chromosomes. Screening a population of 466 BC_2_F_1_ and BC_2_F_2_ individuals, derived from backcrosses of wheat-SWG amphiploid to wheat, by the SWG-specific markers led to selection of 72 plants putatively carrying one or two SWG chromosomes. The genome painting analysis of the 72 plants eventually identified a set of 37 wheat-SWG chromosome addition lines covering all the 14 pairs of SWG chromosomes and two compensating Robertsonian translocations (RobTs). While the wheat-SWG chromosome addition lines and RobTs are invaluable genetic resources for wheat improvement via chromosome engineering, our results showed the power of genome-specific markers in combination with genome painting in dissection of a polyploid genome and implicated the origin of a group of important polyploid grasses.

**Supplementary Information:**

The online version contains supplementary material available at 10.1007/s00122-025-05021-8.

## Introduction

As one of the most important crops, common wheat or bread wheat (*Triticum aestivum* L.; 2*n* = 6*x* = 42; genomes AABBDD) provides ~ 20% calories and nutrients of our daily diet (Tadesse et al. 2016); as the most widely cultivated and most traded grains, wheat production is facing numerous challenges including the changing climate and the pressure from a growing human population (Asseng et al. 2015). In another aspect, the genetic bottlenecks posed by the polyploidization events and domestication significantly reduced the diversity level in the primary gene pool of wheat, and modern wheat breeding further reduced the genetic diversity in this hexaploid species (Avni et al. 2017; Graybosch and Peterson 2010; Maccaferri et al. 2019; Pont et al. 2019). The narrow genetic background increases the vulnerability of the wheat crop to stress and limits the potential for wheat genetic improvement. To improve wheat productivity and sustainability, we need to increase its genetic diversity and expand its gene pool (Graybosch and Peterson 2010; He et al. 2019). The introduction of genetic variation from the wild ancestors and relatives of wheat played an important role in broadening the wheat genetic basis (Cheng et al. 2019; He et al. 2019; Zhou et al. 2020) in addition to targeted improvement of specific traits such as disease resistance (McIntosh et al. 2008).

The genus *Thinopyrum* Á. Löve contains 15 species within the Triticeae tribe (Govaerts et al. 2021), constituting an important resource of the secondary and/or tertiary gene pool of wheat. Many disease-resistant genes have been transferred to wheat from tall wheatgrass (TWG; *Th. ponticum* (Podp.) Barkworth & D. R. Dewey) and intermediate wheatgrass (IWG; *Th. intermedium* (Host) Barkworth & D. R. Dewey) (McIntosh et al. 2008). By contrast, there is little effort for alien introgression into wheat from the remaining 13 species except for *Th. junceiforme* (Á. Löve & D. Löve) Á. Löve, commonly known as sea wheatgrass (SWG). SWG originated in the western European, Mediterranean, Atlantic and Baltic coasts (Heyligers 2006; Hilton et al. 2007; Niento-Lopez et al. 2000) and invaded into Australian coasts (Hanlon and Mesgaran 2014). As a tetraploid (2*n* = 4*x* = 28) species, SWG carries the J_1_ and J_2_ genomes (Dewey 1984), which are believed to be closely related to the J genome of *Th. bessarabicum* or the E genome of *Th. elongatum* (Liu and Wang 1992). SWG was first used as a source for breeding durum wheat (*T. turgidum* L. subsp. *durum* (Desf.) Husn.) with resistance to Fusarium head blight (FHB) (Jauhar and Peterson 2001) and seven prevailing races of stem rust pathogen *Puccinia graminis* Pers.:Pers. f. sp. *tritici* Eriks (*Pgt*) (Turner et al. 2013). We recently developed an amphiploid from a cross between SWG and cultivated emmer (*T. turgidum* ssp. *dicoccum* (Schrank ex Schübl Thell.). Compared to its emmer parent, the amphiploid showed many useful traits, including solid stem for resistance to wheat stem sawfly (*Cephus cinctus* Norton) and resistance to wheat streak mosaic virus (WMSV), stem rust, and FHB. It showed tolerance to abiotic stresses such as waterlogging conditions, low nitrogen supply, and tolerance to salinity (Li et al. 2019). Thus, SWG can be an invaluable source for wheat genetic improvement and can serve to broaden the genetic base of the wheat crop.

As the first step to transfer the agriculturally important traits from SWG into wheat, we are dissecting the SWG genome by developing the wheat-SWG chromosome addition lines via recurrent backcrossing to wheat and selecting the plants carrying one SWG chromosome. In the genomics era, alien chromosome can be detected and traced more effectively by molecular markers (Sharp et al. 1989; Tiwari et al. 2014) and subsequently by fluorescence genomic in situ hybridization (GISH) or genome painting (Schwarzacher et al. 1992) as compared to the traditional but low-throughput cytogenetic techniques, such as meiotic pairing (Sears 1956, 1972) and chromosome banding (Gill and Kimber 1977; Lukaszewski 2000). As an untapped genetic resource, there is little genetic and genomic resources available in SWG for marker development. Furthermore, the lack of a clear picture of its tetraploid genome constitution made it difficult to discriminate the chromosomes of the two sub-genomes by GISH. To this end, we established a new GISH procedure to discriminate the SWG chromosomes of its two sub-genomes, sequenced the SWG genome, and developed 127 SWG-specific markers from the draft assembly. Screening large backcrossing populations using the SWG-specific markers identified plants carrying one or two SWG chromosomes, and GISH analysis of these plants led to identifying a set of 14 wheat-SWG chromosome addition lines. Our results showed that the combination of marker genotyping and GISH analysis is a powerful, genomics-enabled approach to dissect the complex, polyploid genome of a wild relative and laid a foundation for introgression of SWG chromatin into the wheat genome and also implicated the evolution of polyploid *Thinopyrum* species.

## Materials and methods

### Plant materials

A complete wheat-SWG amphiploid 13G819 (2*n* = 8*x *= 56) was developed from a cross between emmer wheat line 13G139 and *Th. junceiforme* accession PI 414667, and two partial amphiploids 14F3516 and 14F3536 (2*n* = 6*x* = 42) were developed from a cross between durum wheat cultivar Afuwan (PI 634318) and PI 414667 (Li et al. 2019). The amphiploids 13G819, 14F3516, and 14F3536 were crossed to Brick, HR58, or Louis, and the F_1_ hybrids were backcrossed to the wheat parents or self-pollinated to develop large populations segregating for the SWG chromosomes. For the present study, 433 plants of BC_2_F_2_ generations and 33 BC_2_F_1_ plants were screened. Their progenies, i.e., the selected BC_2_F_3_ and BC_2_F_2_ (waterlogging tolerance), were characterized by GISH and validated by all markers. The BC_2_F_1_ plants were selected from a waterlogging tolerance assay as described by Li et al (2019). Seeds of *Th. junceiforme* (PI 414667), *Th. bessarabicum* (PI 531711), and *Th. elongatum* (PI 531718) were provided by Dr. David Stout of USDA-ARS (Pullman, WA), and seeds of wheat inbred line HR58 and wheat cultivars Brick, Chinese Spring (CS), and Louis were obtained from Drs. James Anderson (University of Minnesota, St. Paul, MN), Karl Glover (South Dakota State University, Brookings, SD), Bikram S. Gill (Kansas State University, Manhattan, KS), and Kim Kidwell (Washington State University, Pullman, WA).

### SWG genome sequencing and assembly

Genomic DNA was extracted and purified from leaves of PI 414667 using QIAGEN Genomic-tip 500/G (Qiagen, Valencia, CA) following the manufacturer’s instruction. The SWG genomic DNA was used to prepare one paired-end library and two mate-pair libraries with fragment size of 2 kb and 5 kb, and these three libraries were sequenced on the HiSeq platform (Illumina, San Diego, CA) under a commercial service. The quality of sequence reads was assessed using FastQC (Andrews 2010). Preprocessing of reads was conducted using Trimmomatic (Bolger et al. 2014) and Prinseq (Schmieder and Edwards 2011). *K-*mer analysis was conducted using *k*-mer-genie (Chikhi and Medvedev 2013). De novo assembly of paired-end reads was done using ABySS assembler (Simpson et al. 2009).

### Marker development and silico mapping

The SWG contigs were retrieved via a standalone BLAST server using unique query sequences from the mapped wheat EST database (Qi et al. 2004; https://wheat.pw.usda.gov/westsql/index.html), wheat genome assembly (International Wheat Genome Sequencing Consortium 2018), microsatellite/simple sequence repeat (SSR) and high-confidence CDS of *Aegilops tauschii*, the D-genome donor of common wheat (Luo et al. 2017). The retrieved SWG sequences were aligned with their wheat homoeologs using the computer program multiple sequence comparison by log-expectation (MUSCLE; https://www.ebi.ac.uk/Tools/msa/muscle/) to identify indels or Oligonucleotide polymorphisms (ONPs). The alignments were fed to the genome-specific primer tool (Wang et al. 2016) (https://probes.pw.usda.gov/GSP/) to design SWG-specific primers targeting the indel or ONP variations at the 3′ end of the primers. Primer 3 (Untergasser et al. 2012) was also used to pick the universal primers from the SWG sequence for combining with the SWG-specific primers. The validated SWG-specific markers were anchored to the D-genome chromosomes of *A. tauschii* based on locus homology on a hypothesis that the J_1_ and J_2_ genomes of *Th. junceiforme* are collinear with the D-genome. The sequences of the primers, amplicon size, origin of SWG chromosomes, and PCR parameters are listed in Table S1.

### Genome in situ hybridization (GISH)

The GISH characterization of the SWG chromosomes in the wheat backgrounds was performed following the protocol described by Li et. al. (2019) with modification for discriminating the J_1_ and J_2_ genomes. The genomic DNA from *Th. elongatum* accession PI 531718 was labeled with biotin-16-dUTP as the probe to detect J_1_ genome, while the genomic DNA from accessions TA 2140 and TA 2196 of *D. villosum* (L.) Coss. & Durieu ex Candargy [syn. *Haynaldia villosa* (L.) Schur, 2*n* = 2*x* = 14, VV] was labeled with digoxigenin-11-dUTP to detect J_2_ genome in 13G 819 and its derivatives. BIO-PROBE® Nick translation DNA labeling system (Enzo Biochem, Inc, New York, NY) was used in this nick translation following the manufacturer’s instruction. The wheat chromomeres were counterstained with 4′,6-Diamidine-2′-phenylindole dihydrochloride (DAPI). Chromosome spread preparation, GISH, microscopy, photography, and GISH image analysis were conducted by following the protocols described by Li et. al. (2019).

### Genotyping

Genomic DNA was isolated from the plant leaves according to Li et. al (Li et al. 2008) with modifications using an extraction buffer containing cetyltrimethylammonium bromide (Sigma-Aldrich, St. Louis, MO). A 15 µL polymerase chain reaction (PCR) was set up containing ~ 100 ng of genomic DNA, 0.2 µM primers, 1 × GoTaq® buffer (Promega, Madison, WI), 0.25 mM dNTPs, and 1 unit of *Taq* polymerase. The PCR was conducted on a GeneAmp 9700 or Veriti thermocycler (ThermoFisher Scientific, Waltham, MA). PCR profile consisted of initial denaturation for 4 min at 94 °C, 35 cycles of 30 s at 94 °C, 30 s at 51–59 °C, 55–60 s at 72 °C, and 7 min of final extension at 72 °C. The PCR products greater than 300 bp were separated by electrophoresis in the 3% agarose gel, and for amplicons smaller than 300 bp were analyzed by electrophoresis in 6% polyacrylamide gel. The gel was stained using ethidium bromide and viewed under UV light using the U: GENIUS Gel documentation system (SDI Group, UK). Genomic DNA of *D. villosum* accession GP005 was from our previous study (Li et al. 1995).

### Hierarchical clustering of marker groups

The marker scoring data matrix was filtered to remove rows (samples) and columns (markers) that consisted of negative result for all entries. The presence of the marker in a sample was scored as ‘1’, absence as ‘0’. Analysis was conducted using R programming (R Core Team 2013). An R library, ‘vegan’ (version 2.7–1) was used to calculate the distance matrix (Oksanen et al. 2025). Function ‘veglist’ with ‘method’ =  ‘jaccard’ computed the distance matrix. Hierarchical clustering of markers was then conducted using ‘hclust’ function with the argument ‘method’ = ‘average’.

## Results

### A draft assembly of the SWG genome

Genome sequencing generated 3,103,558,240 reads, of which 2,716,213,250 reads were from the paired-end library, 197,419,161 and 189,925,829 reads were from the two mate-pair libraries. The ~ 2.7 billion paired-end reads were assembled into the first SWG draft assembly of ~ 10 Gb with > 24 million contigs with a N50 of 433 bp (Table 1). Of the 24 million contigs, ~ 1.3 million are longer than 1 kb and were used for marker development. We are improving the SWG genome assembly by integration of the mate-pair reads and using the long-read technologies, and detailed analysis of the improved SWG genome assembly will be published separately.

**Table 1:** Assembly of paired end reads with ABySS (40x)

Number of contigs > 200 bp	24,286,735
Size of the assembly	10,260,414,562 bp
Longest contig	39,988 bp
Number of contains > 1 Kb	1,294,244
Number of contains > 10 kb	3355
N50	435 bp

### Development of SWG-specific markers

Three types of queries were used to retrieve SWG contigs for marker development: (1) mapped single-copy ESTs, which were mapped to all A-, B-, and D-genome chromosomes of the seven homologous groups (https://wheat.pw.usda.gov/westsql/index.html),(2) *A. tauschii* microsatellite-containing genes, and (3) *A. tauschii* high confidence coding sequence (CDS; Luo et al. 2017). A total of 202 pairs of primers were designed, including 56 pairs from the contigs retrieved by ESTs, 68 pairs from the contigs using microsatellite-containing genes, and 78 pairs from the contigs retrieved by CDS. Finally, we developed four markers using sequences of genes with known function as queries, two from the *PinA* (Gautier et al. 2000), one from the *EXPB1* (Liu et al. 2007), and one from the *TthV* (Castagnaro et al. 1992). In addition, 14 markers were adopted from two distant relatives of wheat, *D. villosum* and *Leymus* (Ali et al. 2016; Cao et al. 2009; He et al. 2013; Kaur et al. 2008; Zhang et al. 2018). As a result, a total of 220 markers were designed.

**Fig. 1:**
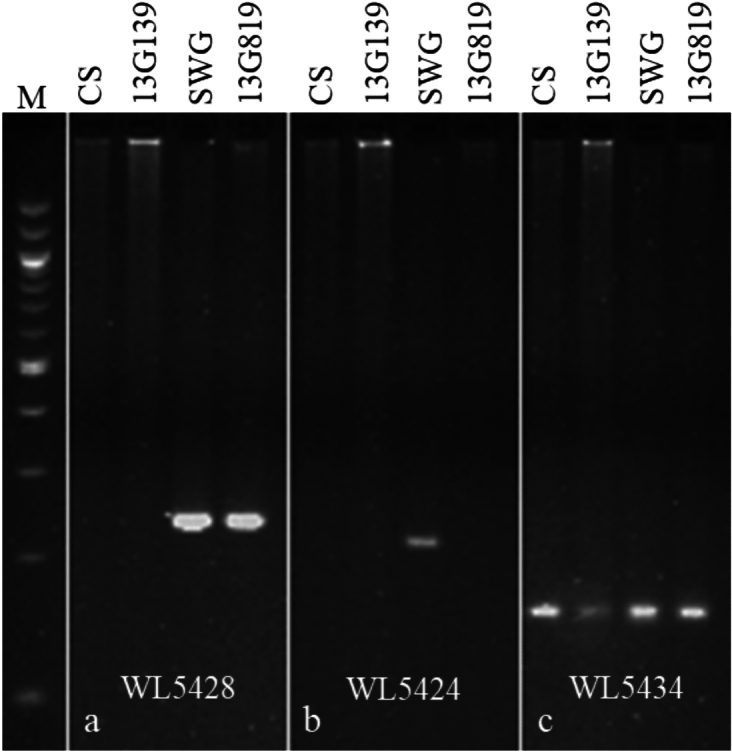
Validation of genome specificity of the designed markers. **a** Marker is specific for SWG and amphiploid. **b** Marker is specific for SWG. **c** Marker is monomorphic for all parents. The DNA samples are indicated on the top, CS, 13G139 (emmer parent), SWG (accession PI 414667), and 13G819 (emmer-SWG amphiploid). Markers are indicated at the bottom of the figure. Marker (M) at the left side of the figure is a 100 bp ladder

We examined the genome specificity of the 220 markers by representative genotyping of the CS, 13G139 (emmer parent), SWG accession PI 414667, and 13G819 (amphiploid). The results showed that 140 markers specifically amplified from SWG, of which 127 markers amplified a fragment from both SWG and amphiploid (Fig. 1a), and 13 markers amplified fragments specifically from the SWG DNA but did not amplify a fragment from the amphiploid (Fig. 1b). The remaining 87 markers detected monomorphisms between wheat and SWG (Fig. 1c).

Of the 127 SWG-specific markers, 22 markers were designed using the EST-based pipeline, 50 using the *A. tauschii* high confidence CDS as queries, 48 using the *A. tauschii* microsatellite-containing genes as queries, and one marker using *PinA* sequence. The remaining six markers were transferred from *D. villosum* (He et al. 2013) and *Leymus* (Kaur et al. 2008). Out of 127 markers, 108 are dominant and amplified a fragment only from SWG, and the remaining 19 are co-dominant, which amplified polymorphic fragments from the SWG and wheat genomes.

**Fig. 2:**
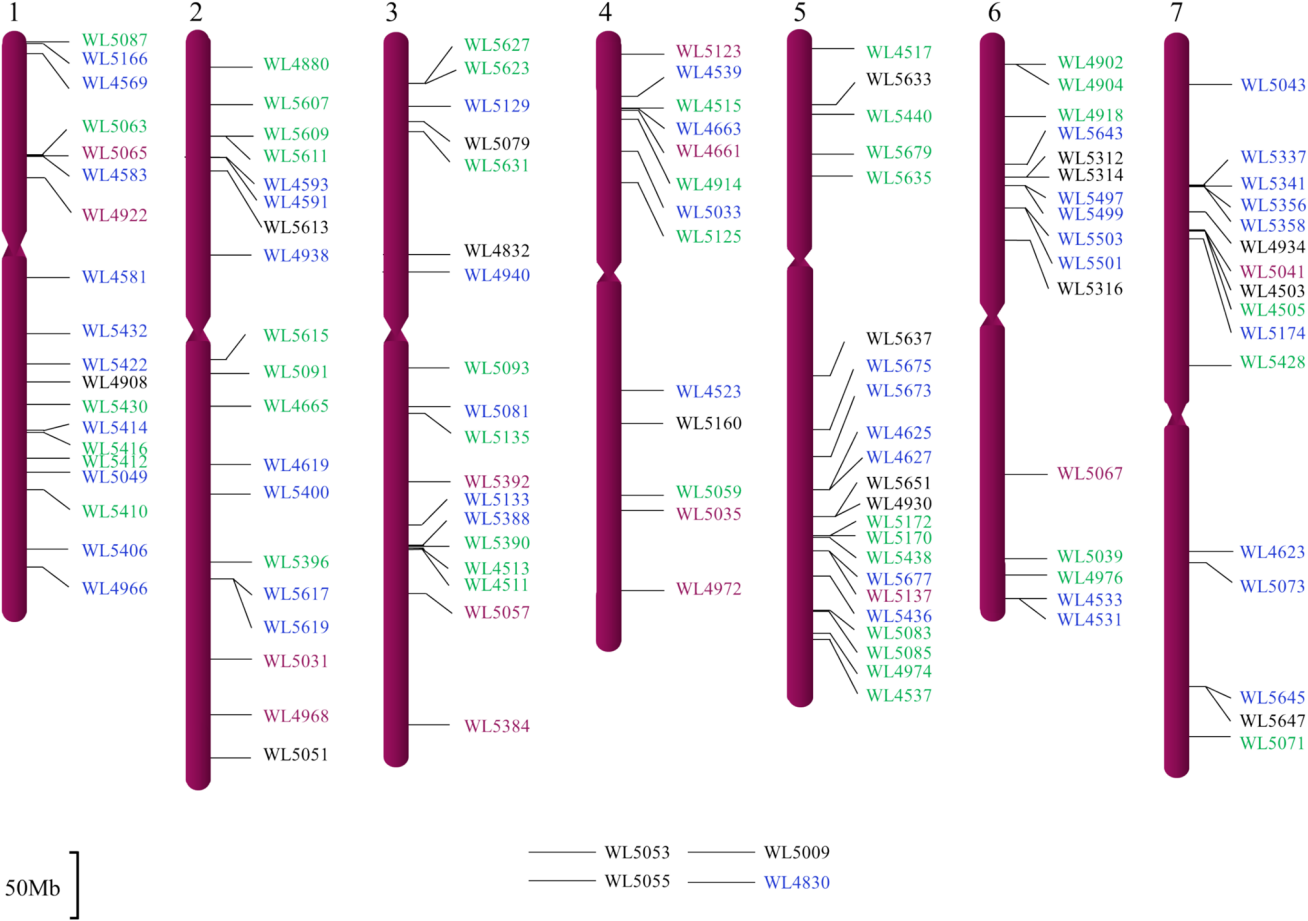
Anchoring the SWG-specific markers to the D-genome chromosomes of *Aegilops tauschii*. The homoeologous groups are indicated at the top of the maps, and marker loci are indicated at the right of the maps, and their position on the D-genome chromosomes are indicated the black bars. The markers in green are positive for the J_1_ genome, the markers in blue are positive for the J_2_ genome, the markers in purple are co-dominant for both the J_1_ and the J_2_ genomes as assayed in the wheat-SWG addition lines, and the markers in black are not assigned to a particular genome. Four markers at the bottom were not assigned to any position because WL5053, WL5055, and WL5009 are specific to multiple locations on SWG genome and WL 4830 was designed from a chromosome 3 sequence of *A. tauschii,* but in SWG genome it was specific to 5J_2_chromosome, however its location on 5J_2_ is unknown. The scale bar in the bottom left corner indicates 50 Mb

We assigned 127 SWG-specific markers on individual SWG chromosomes and placed them to the 7 chromosomes of *A. tauschii*, the core genome of Triticeae species, based on sequence homology via BLASTn searches of its annotated genome (Fig. 2). We found that the genic sequences of wheat or *A. tauschii* have identity ranging from 87 to 98% when aligned to the SWG genome.

We randomly selected 32 SWG-specific markers and applied them to the DNA of *Th. bessarabicum*, *Th. elongatum*, and *D. villosum*. Out of the 32 markers, 21 were positive for *Th. bessarabicum*, 10 markers were positive for *D. villosum*, and nine markers were positive for *Th. elongatum*. Only two markers were specific to all three diploids species and *Th. junceiforme* (Table S2). This result represents the variation among the species closely related to SWG and the transferability of markers designed to the SWG’s related species.

### Chromosome-based dissection of the SWG genome

**Fig. 3:**
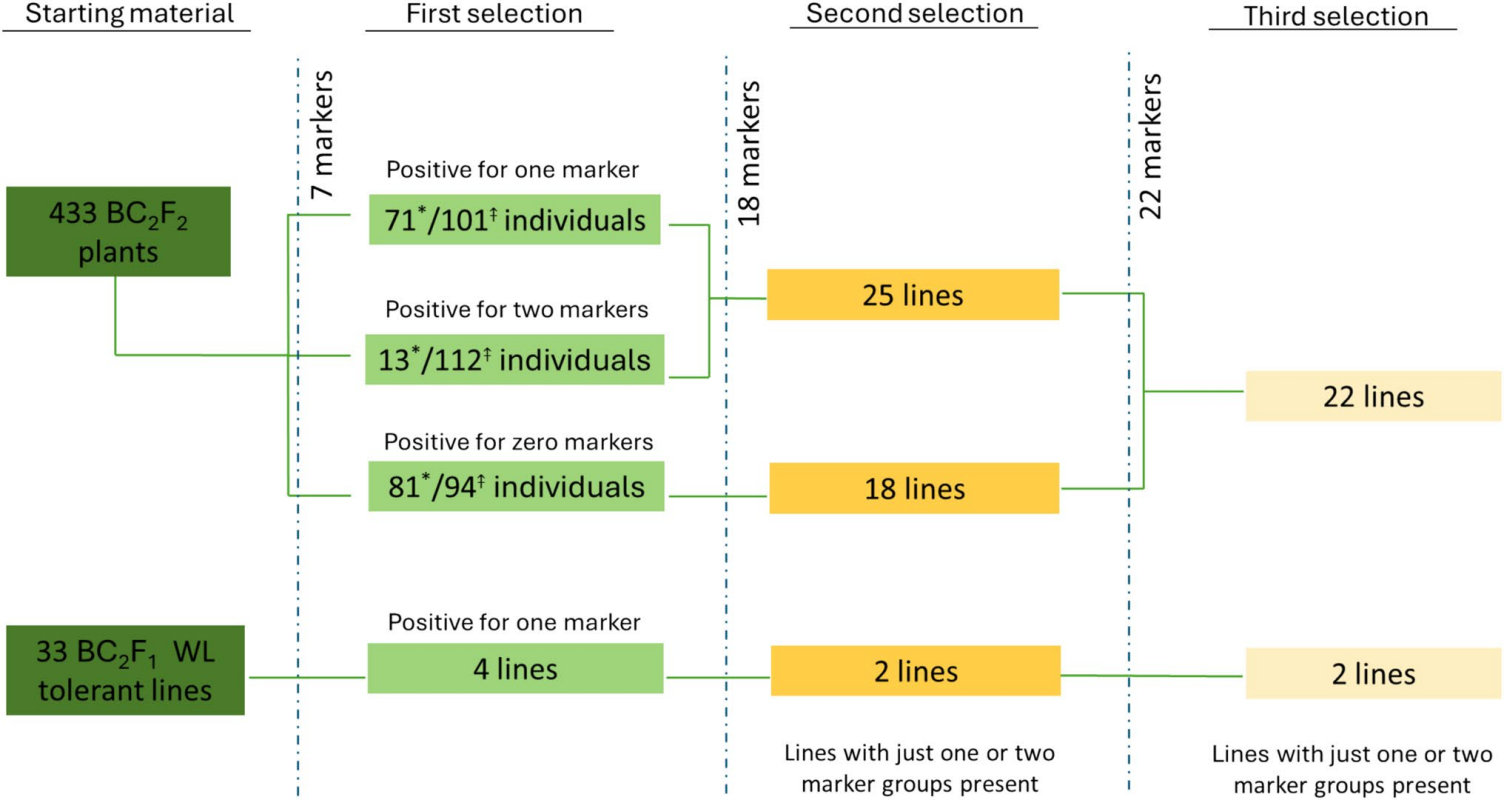
Flowchart depiction of addition line selection strategy. Starting material of 433 BC_2_F_2_ individuals and 33 BC_2_F_1_ waterlogging (WL) tolerant lines given on the left most part. Three stages of marker screenings are depicted by dashed blue lines and annotated with the number of markers used in each screen. ⤉ The total individuals positive for the respective number of markers. *The number of lines selected for subsequent marker screening

We screened 466 BC_2_ plants, including 33 BC_2_F_1_ selected from waterlogging tolerance assay and 433 BC_2_F_2_ individuals, using seven SWG-specific markers, each of which was specific to a distinct homoeologous chromosome group (Fig. 2). A flowchart depicting the strategy and procedure for marker-based addition line selection is shown in Fig. 3, and three batches of markers are listed in Table S3. In the initial screening, the number of markers positive in a plant sample ranged from zero to six (Fig. S1). Of the 466 plant DNA samples, 94 BC_2_F_2_ were negative for all seven markers, four BC_2_F_1_ and 101 BC_2_F_2_ were positive for one marker, and 112 BC_2_F_2_ were positive for two markers (Fig. S1). The plants with zero, one, or two positive markers were subjected to a second round screening using additional markers. Meanwhile, we reduced the size of the BC_2_F_2_ populations by eliminating potential duplicates based on the BC_2_F_1_:F_2_ pedigree. We selected 169 plants, including 81 zero-marker plants, 71 one-maker plants, 13 two-marker plants, and four waterlogging tolerant plants, for a second round screen with 18 new markers (Fig. 3; Table S3). The second screening further narrowed the population to 45 DNA samples carrying markers from one or two homoeologous groups (Fig. 3). However, the small number of markers made it infeasible to partition markers between two sub-genomes. Thus, these 45 DNA samples were subjected to a third round screen with 22 new markers (Table S3). Hierarchical clustering of markers using their presence/absence data among samples clustered markers into fourteen groups representing fourteen SWG chromosomes, and markers from the same homoeologous groups that clustered separately were referred to as J_x_ and J_y_ specific markers before their origin of sub-genomes was revealed via GISH (Fig. S2; Table S3). Among fourteen groups, distinction of 3J_y_ from 7J_x_ and 5 J markers between 5J_x_ and 5J_y_ was relatively ambiguous, due to fewer chromosome specific markers (Fig. S2). Nevertheless, 14 tentative groups were assumed to shortlist putative addition lines, resulting in a list of 24 lines carrying markers from one or two homoeologous groups as putative wheat-SWG addition lines for GISH characterization (Fig. 3).

### GISH characterization of putative wheat-SWG addition lines

**Fig. 4:**
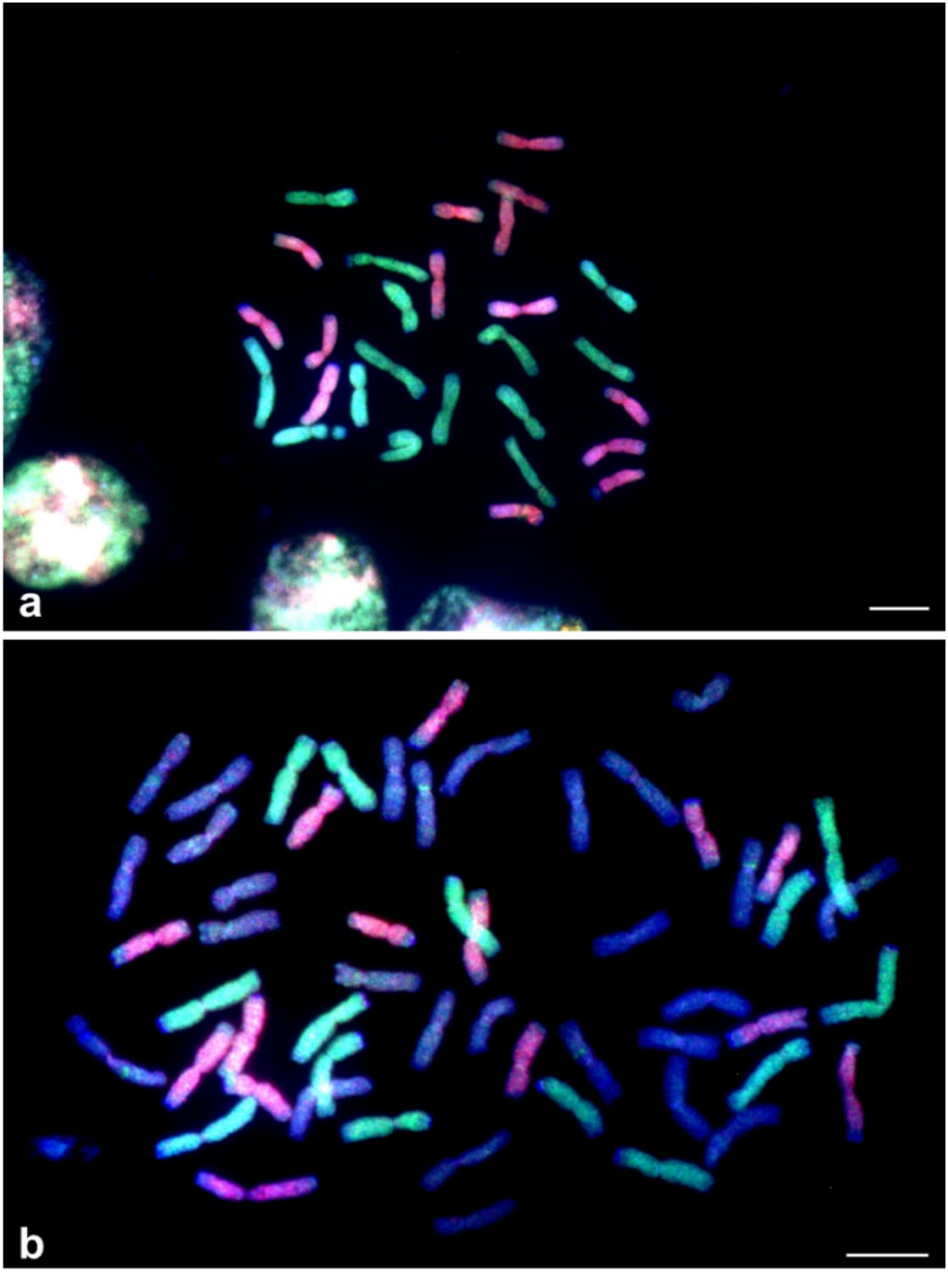
GISH discrimination of the J_1_ and J_2_ chromosomes. The 14 J_1_ genome chromosomes were painted in green by biotin-labeled genomic DNA of *Th. elongatum*, and the 14 J_2_ genome chromosomes were painted in pink by digoxigenin-labeled genomic DNA of *D. villosum*, in *Th. junceiforme* (SWG) **a** and in the emmer wheat-SWG amphiploid 13G819 **b** The 28 wheat A- and B-genome chromosomes were counterstained in blue or purple by 4′,6-Diamidine-2′-phenylindole dihydrochloride (DAPI) in the amphiploid **b** Bar = 10 µm

Using the multi-color GISH (mcGISH), we previously demonstrated that SWG is an allotetraploid with its J_1_ genome closely related to *Th. bessarabicum* and *Th. elongatum*, and its J_2_ genome was derived from an unknown source (Li et al. 2019). Considering that a significant portion of SWG-specific markers hit the V genome of *D. villosum* (Table S2), we included *D. villosum* as source DNA to distinguish the J_1_ and J_2_ genomes in addition to *Th. bessarabicum* and *Th. elongatum*. Using the fluorescence-labelled DNA of diploid sources of *Th. elongatum* and *D. villosum*, we clearly distinguished the J_1_ from J_2_ genome in SWG (Fig. 4a) and wheat-SWG amphiploid (Fig. 4b). Among 56 chromosomes in the wheat-SWG amphiploid 13G819, the 14 J_1_ genome chromosomes were painted in green by biotin-labeled genomic DNA of *Th. elongatum*, the 14 J_2_ genome chromosomes were painted in pink by digoxigenin-labeled genomic DNA of *D. villosum*, and the remaining 28 wheat A- and B-genome chromosomes were counterstained in blue or purple by DAPI (Fig. 4b). This result suggests that one of the SWG genome (J_1_) is related to the *Th. elongatum* E genome and another one, the J_2_ genome, to the V genome of *D. villosum.*

**Fig. 5:**
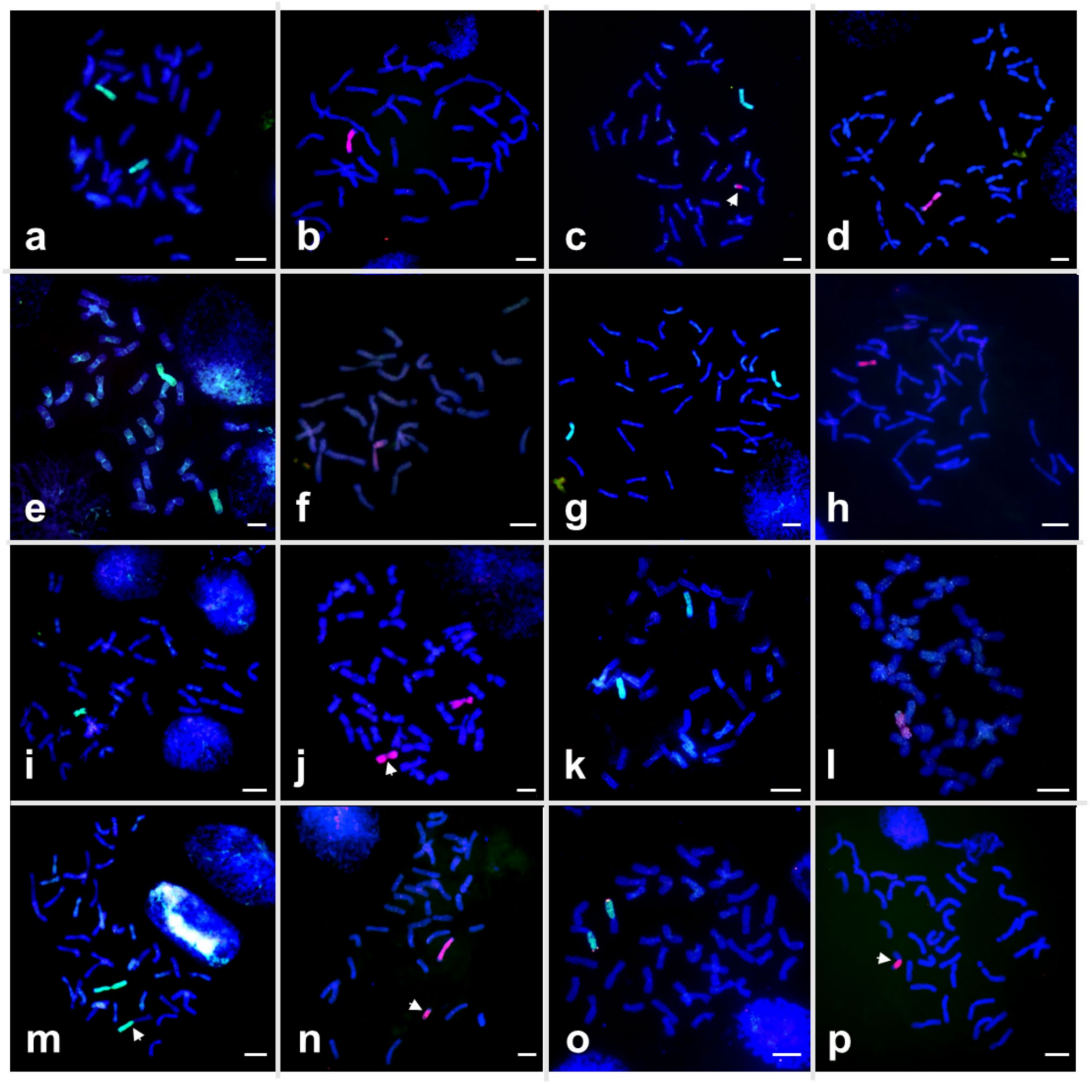
Multi-color GISH analyses of wheat-SWG chromosome lines. **a** DA1J_1_, **b** MA1J_2_, **c** MA2J_1_ plus a W-J_2_ RobT (arrow), **d** MA2J_2_, **e** DA3J_1_, **f** MA3J_2_, **g** DA4J_1_, **h** MA4J_2_, **i** MA5J_1,_ **j** DA5J_2_ plus a J_2_ isochromosome (arrow), **k** DA6J_1_, **l** MA6J_2_, **m** MA7J_1_ plus a W-J_1_ RobT (arrow), **n** MA7J_2_ plus a J_2_ telocentric chromosome (arrow), and **o** W-3J_1_L RobTs, and **p** W-4J_2_ RobT. The J_1_ genome chromosomes were painted in green by biotin-labeled genomic DNA of *Th. elongatum*, the J_2_ genome chromosomes were painted in pink by digoxigenin-labeled genomic DNA of *D. villosum*, and the wheat chromosomes were counterstained in blue or purple by DAPI. The scale bars indicate 10 µm

After the second round of marker screening, we submitted seeds of 72 lines (68 selected based on the incomplete genotyping data and four lines from waterlogging tests) to the Xu Lab for mcGISH characterization. Luckily, the final set of 24 lines carrying one or two SWG chromosomes based on the 47 markers reported above were included in the 72 samples of seeds. The mcGISH results validated the marker-selected plants with identification of 37 wheat-SWG addition lines, including eight disomic addition (DA) lines, 22 monosomic addition (MA) lines, and 7 double monosomic addition (DMA) lines (Table 2). Furthermore, mcGISH determined the genome origin of the SWG chromosomes in the addition lines (Fig. 5). Combining the homoeologous chromosome group assignment to the addition lines by marker genotyping, we identified DA lines for six SWG chromosomes and MA lines for 11 SWG chromosomes in addition to the DMA lines (Fig. 5 and Table 2).

**Table 2:** Addition lines for each chromosome group and their marker characterization summary

Chromosome group	Lines	Total markers	Positive markers	Type of addition line*	Samples in Fig. 5
1J_1_	F18-54	6	6	DA	Fig. 5a
	F18-56	6	5	DMA	
1J_2_	F18-3	10	10	MA	Fig. 5b
	F18-98	10	4	MA	
	F18-7	10	4	MA	
2J_1_	F18-8	7	6	MA + W-J_2_	Fig. 5c
	F18-2	7	3	DMA	
	F18-4	7	3	DMA	
	F18-5	7	3	DMA	
2J_2_	F18-90	7	6	MA	Fig. 5d
3J_1_	F18-53	10	10	DA	Fig. 5e
	F18-23	3	2	MA	
	F18-51	3	3	DMA	
3J_2_	F18-57	7	7	MA	Fig. 5f
4J_1_	F18-99	7	7	DA	Fig. 5g
	F18-101	7	7	DA	
	F18-103	7	7	MA	
	F18-102	7	6	MA	
	F18-112	7	5	MA	
	F18-114	7	5	MA	
	F18-116	7	5	DMA	
4J_2_	F18-73	8	6	MA	Fig. 5h
	F18-119	8	6	MA	
	F18-105	8	3	MA	
5J_1_	F18-39	9	9	MA	Fig. 5i
	F18-41	9	9	MA	
	F18-38	6	4	DA	
	F18-40	6	4	DA	
5J_2_	F18-19	7	5	MA + iJ_2_	Fig. 5j
	F18-59	7	3	MA	
6J_1_	F18-81	5	5	DA	Fig. 5k
	F18-80	5	3	MA	
6J_2_	F18-97	4	4	MA	Fig. 5l
	F18-96	4	2	DMA	
7J_1_	F18-84	3	3	MA + W-J_1_	Fig. 5m
	F18-85	3	3	DA	
7J_2_	F18-62	10	10	MA + tJ_2_	Fig. 5n

*MA monosomic addition, *DA* disomic addition; and *DMA* double monosomic addition

### Validation of wheat-SWG addition lines with markers

After identification of addition lines via GISH, we characterized the 37 wheat-SWG chromosome addition lines using SWG-specific markers. A total of 100 markers spanning all fourteen chromosomes were used for the characterization of 37 lines (Table 2). These 100 markers included the 47 previously used markers as described above (Fig. 3; Table S3) and 53 newly designed markers. Of the 100 markers, 94 were positive in the addition lines (Table 2). Apart from 2J_1_, 2J_2_, 4J_2_, and 5J_2_, all chromosomes had at least one line positive for all markers specific for that chromosome (Table 2). This set of addition lines was then used to determine the sub-genome specificity of the remaining 27 markers, 13 of which were positive among the corresponding addition lines’ DNA samples. Thus, marker characterization of addition lines not only validated the identity of the added SWG chromosomes but also assigned 107 out of 127 total markers to the specific SWG chromosomes (Table S1). Of the 107 markers, 45 were specific to the J_1_ genome, 48 were specific to the J_2_ genome, and 14 were specific to the homoeologous chromosomes of both J_1_ and J_2_ genomes (Fig. 2 and Table S4). The remaining 20 markers have not been assigned to a particular genome. The chromosomal location and genome origin of these markers is indicated in Fig. 2. Based on the marker genotyping data, we have obtained at least one addition line for each of the 14 SWG chromosomes (Table 2).

### Whole-arm translocation and SWG-derived phenotypes

Besides the wheat-SWG addition lines, we also identified telosomics of SWG chromosomes and Robertsonian translocations (RobTs), including four J_1_ genome telocentric chromosomes, 15 J_2_ genome telocentric chromosomes, six J_1_-J_2_ RobTs, three wheat (W)-J_1_ RobTs, and 10 W-J_2_ RobTs. Some wheat-SWG RobT lines showed good fertility, indicative of genetic compensation. These include a disomic W-3J_1_L RobT line (Fig. 5o) and a monosomic W-4J_2_ RobT (Fig. 5p). A monosomic W-7J_1_L RobT (Fig. 5m), a monosomic W-2J_2_ RobT (Fig. 5c), and J_2_ isochromosome (Fig. 5j), and J_2_ telocentric chromosome (Fig. 5n) were present in the wheat-SWG addition lines. The presence of the isochromosome, telosomics, and RobTs suggests that mis-division of univalent chromosomes is highly active in wheat-SWG progenies.

**Fig. 6:**
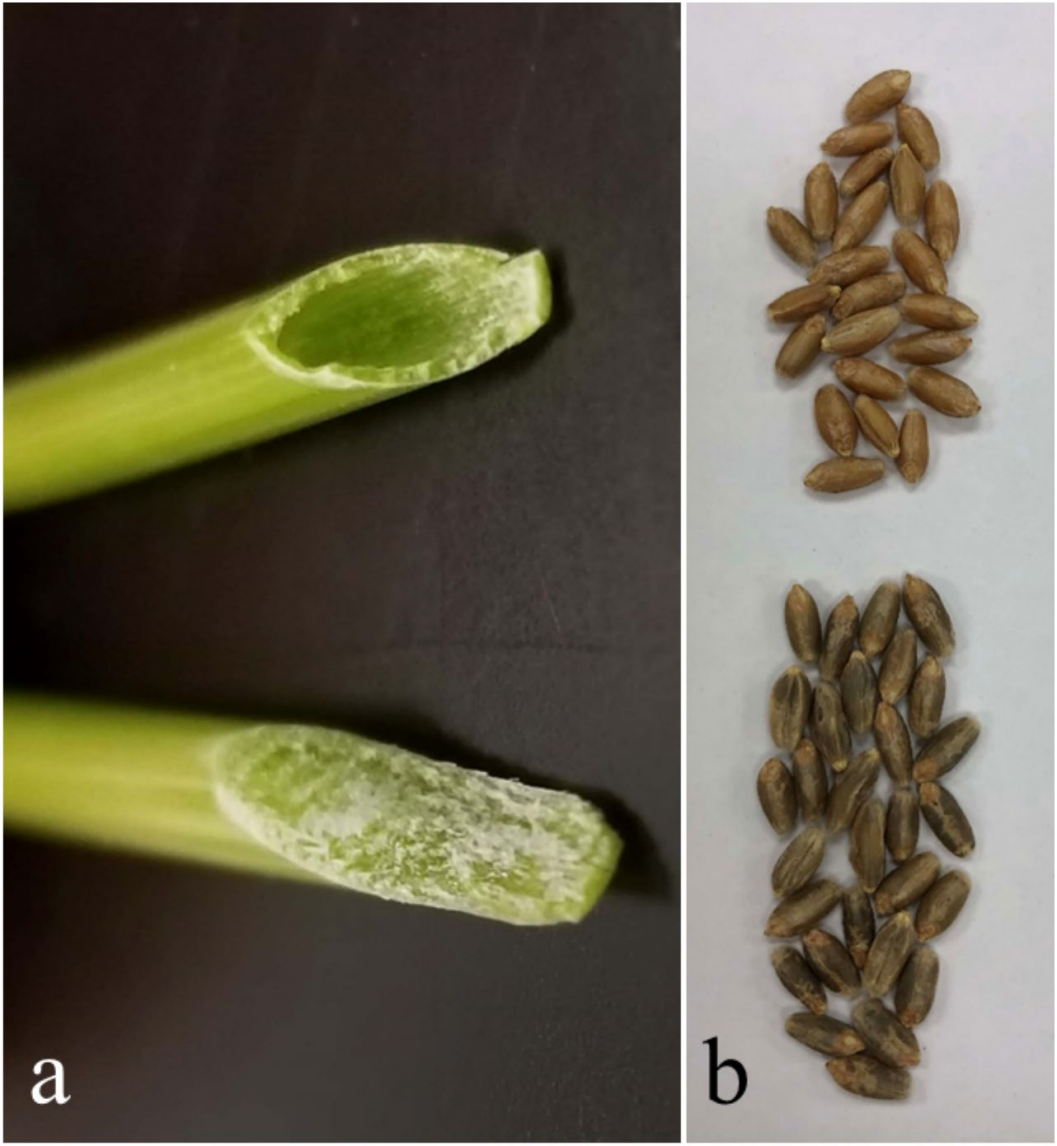
Location of morphological traits to SWG chromosomes. **a** Cross sections of stems of wheat segregant (top) and DA3J_1_ (bottom). **b** Grains of wheat segregant (top) and DA4J_1_ (bottom). The wheat segregant plants (top) showed no amplification for SWG-specific markers

Phenotyping the addition lines localized SWG-originated morphological traits to the SWG chromosomes. DA3J_1_ (Fig. 6a) and W-3J_1_L RobT showed solid stem, indicating the gene for stem solidness is located on 3J_1_L, and DA4J_1_ produced blue-colored seeds (Fig. 6b), localizing the gene to this SWG chromosome.

## Discussion

SWG is an important gene resource for improving wheat resistance to diseases, insects, and abiotic stress. The progress to transfer these agriculturally important traits to wheat has been slow. The major obstacles are the knowledge gap in understanding of origin of its J_1_ and J_2_ genomes and lack of genomic resources. In the present research, we sequenced the SWG genome and developed a draft assembly, which helped the development of SWG-specific markers. Combining the marker genotyping and genome-specific painting, we dissected the SWG genome into wheat-SWG chromosome addition and RobT lines and accelerated the process for transferring the traits into wheat and shed light to the evolution of this tetraploid species.

### Origin of *Th. junceiforme*

The origin of the J_1_ and J_2_ genomes has been elusive for more than half a century. Meiotic pairing analyses of the F_1_ hybrids between the tetraploid SWG and diploid relatives suggested that both J_1_ and J_2_ genomes are closely related to E genome of *Th. elongatum* and the J genome of *Th. bessarabicum* (Cauderon and Saigne 1961; Jauhar and Peterson 2001; Liu and Wang 1992; McGuire 1984; Pienaar et al. 1988; Wang and Hsiao 1989). One of the major reasons for this scenario was probably because the diploid candidates were selected from the perennial species. Using Oligo-FISH, Qi et al (2023) and Wang et al (2025) found one of the genomes of SWG and intermedium wheatgrass (*Th. intermedium*) is related to *D. villosum*. In the present study, we used mcGISH to analyze the SWG genome and wheat-SWG chromosome lines and corroborated that the J_2_ genome is closely related to the V genome of *D. villosum* via genome painting (Fig. 4), which also gained support from assay of the SWG-specific markers (Table S2). With the availability of high-quality assemblies of the E genome (Wang et al. 2020) and V genome (Zhang et al. 2023), the development of the chromosome-wise assembly of the SWG genome sequences will eventually provide validation of this notion at the sequence level.

### SWG-specific molecular markers

Molecular markers are an important breeding tool in the genomics era. Aligning the SWG genome sequences with their wheat homoeologs, we developed 140 SWG-specific markers. Out of 140 markers, 127 were amplified on amphiploid (13G819) DNA, suggesting that the SWG genome is heterozygous, and 13G819 may not have inherited all the genomic variations present in SWG accession PI414667. Genetic heterozygosity exists extensively in perennial species like SWG. Based on the ratio of markers specific to both amphiploid and sea wheatgrass, ~ 90% of the SWG alleles were captured in the amphiploid 13G819. Otherwise, these marker loci were deleted during the formation of the amphiploid as observed in other amphiploids derived from crosses between the Triticeae species (Feldman and Levy 2012).

The SWG-specific markers facilitated dissection of the SWG genome, and the wheat-SWG chromosome addition lines, in turn, facilitated the assignment of markers to individual SWG chromosomes (Table 2). Of the 107 markers analyzed, 14 went to both J_1_ and J_2_ chromosomes, and the remaining 95 are genome specific, highlighting divergence between J_1_ and J_2_ genomes. We selected query sequences from the *A. tauschii* genome evenly distributed on the seven D-genome chromosomes to retrieve SWG homologs for markers development. When the SWG-specific markers were anchored on the D-genome, however, marker density in the pericentric region is much lower (Fig. 2). This is because most SWG markers are gene-derived, and the proximal regions are gene-poor (Qi et al. 2004).

### Chromosome-scale dissection of the SWG genome and transfer of useful genes

Alien chromosomes or segments in wheat background were traditionally detected by meiotic pairing (Sears 1956, 1972) and chromosome banding (Gill and Kimber 1977; Lukaszewski 2000). These cytogenetic technologies are laborious and have low throughput. While molecular markers can provide homology-based information of the introduced alien chromosomes or segments in a high throughput fashion, GISH provides chromosome context information such as translocation, physical position, and size of alien fragments. In the present study, marker genotyping and GISH karyotyping have been perfectly combined for monitoring SWG chromosomes. We shrank the large BC_2_F_1_ and BC_2_F_2_ populations by low-coverage marker scan and elimination of multi-positive plants and then focused on a small number of progenies for detailed GISH characterization and analysis with additional markers. Furthermore, the mcGISH also facilitates assigning the SWG chromosomes in the addition and RobT lines to the J_1_ and J_2_ sub-genomes.

Phenotyping the addition lines located the genes for solid stem and blue grain color to chromosomes 3J_1_ and 4J_1_, suggesting they are possibly homoeologous to the stem solidness gene(s) on the 3L chromosome arms of wheat (Cook et al. 2004; Lanning et al. 2006) and *Ba1* on the 4el2 chromosome of *Th. elongatum* (Keppenne and Baenziger 1990), respectively. While the W-3J_1_L RobT line can be used as a novel genetic resource for improving wheat resistance to sawflies, the set of addition lines will be screened for resistance to diseases such as FHB and WSMV. For identification of the SWG-derived abiotic stress tolerance, it would be better to use more stable chromosome lines, such as RobT lines with the genetic background cleaned up by backcrossing to a reference wheat cultivar.

## Supplementary information

Below is the link to the electronic supplementary material.

Supplementary file1 (DOCX 1191 kb)

## Data Availability

The datasets generated during and/or analyzed during the current study are available from the corresponding author on reasonable request.
