# Supplementary material for: Genomics-enabled dissection of sea wheatgrass genome for advancing wheat genetic resources
Source: Theor Appl Genet. 2025 Sep 18;138(10):252. doi: 10.1007/s00122-025-05021-8 (PMC12446121; doi:10.1007/s00122-025-05021-8)
Supplement: Supplementary file 1 — Supplementary file1 (DOCX 1191 kb) [file 122_2025_5021_MOESM1_ESM.docx]

Fig. S1. Distribution of the seven markers among the 433 BC_2_F_2_ individuals. The numbers of plants are indicated at the left of the figure, and the numbers of markers per plant are indicated at the bottom of the figure.

**
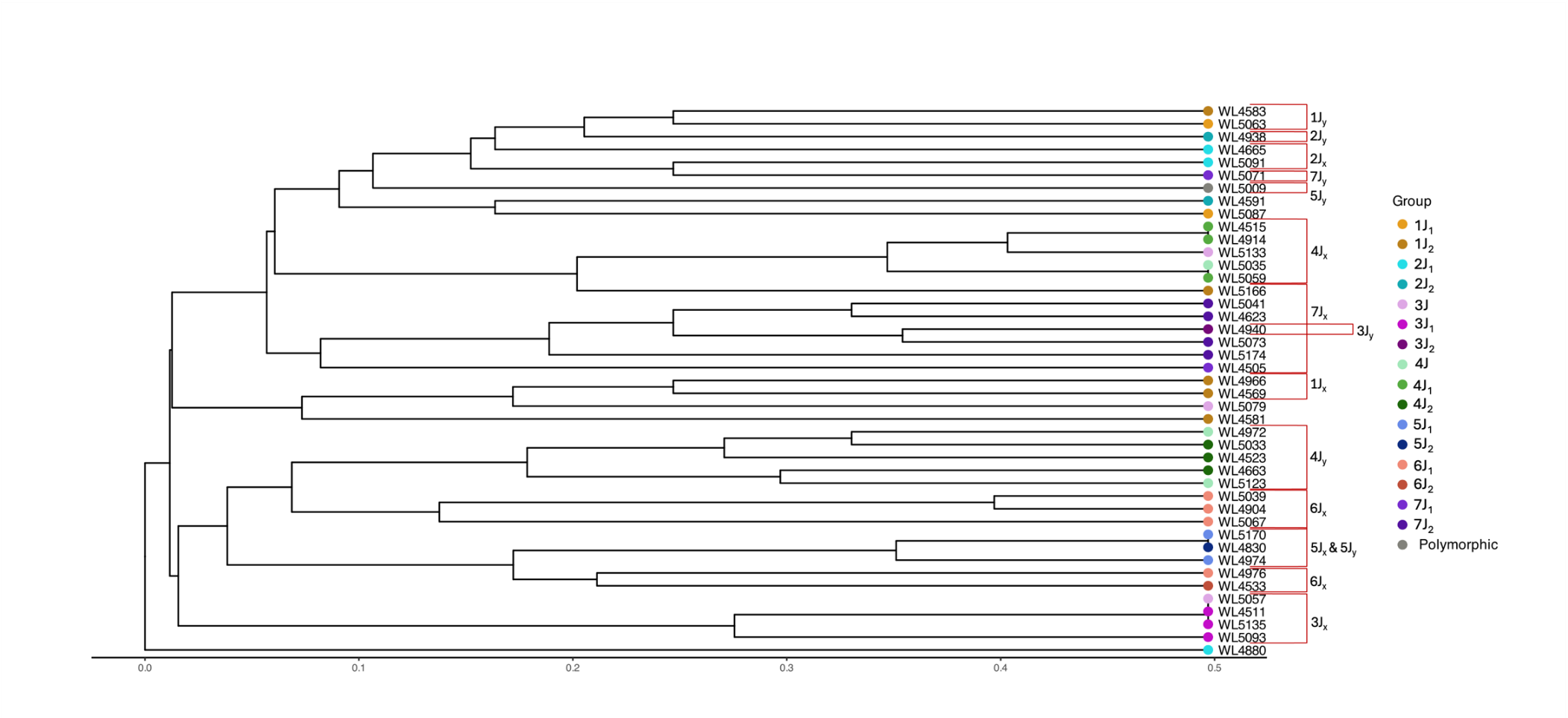
**

**Fig. S2. Hierarchical clustering of 43 markers into fourteen groups corresponding to the fourteen SWG chromosomes.** Clustering is based on the presence/absence data of markers among 42 BC_2_F_2_ samples. The cluster labeling using red lines represents fourteen clusters/groups assumed based on the marker scoring data of 42 BC_2_F_2_ lines. The color key represents true marker specificity obtained after marker characterization using specific addition lines. The WL4940 grouping was ambiguous because of its co-segregation with chromosome 7J_2_ markers. Characterization of markers using SWG addition lines later revealed that WL4940 amplifies a fragment from 3J_2_ as well as 7J_2_ addition line sample. J_x_ and J_y_ was simply used to distinguish two groups/clusters of markers belonging to same homoeologous chromosome group. They don’t represent a single sub-genome. Thus, J_x_ did not always represent J_1_ or vice-versa.

**Table S1. Primer sequences, homoeologous group, product size and annealing temperature of SWG-specific markers.**

| Marker | Forward Primer | Reverse Primer | groups | Product Size (bp) | Annealing temperature (⁰C) |
| --- | --- | --- | --- | --- | --- |
| WL5087 | TGAACTGAAGAGTGCAGAGCAAG | CGAGCGTTCTTTGTATTTGATTT | 1J1 | 317 | 57 |
| WL5166 | GCGATTGGTGCAAGTAAACT | TCATGTTCAGCCCAGATTAGC | 1J2 | 242 | 57 |
| WL4569 | GAGGCAGCACATCTAAAACAGC | GCAGAGAGAAAACCAGCTTCA | 1J2 | 439 | 54 |
| WL5063 | AGAAAAGCCACACACACACA | CGACCCAGAACGACACCAT | 1J1 | 242 | 57 |
| WL5065 | GGACAGCAGGACACAAAGTAAG | TGTTCTTCATTTTACCCCAGTGA | 1J1, 1J2 | 474 | 57 |
| WL4583 | CAAAGAAAAAGCTATCACTGGGT | ATTTGCGTGGCTGGTGAA | 1J2 | 493 | 54 |
| WL4922 | TTTTCTTGTTGCTGCGAGTG | GTCCCTTTCGTCACACCCTA | 1J1, 4J2 | 248 | 57 |
| WL4581 | GCAGGGAGAAATCAAAGAGGAA | TCTAATAAACCGCACGCAAATA | 1J2 | 309 | 54 |
| WL5432 | GATTTCCGCACTAACTCTTTGC | ACCAGTCATGTACAACCATCAA | 1J2 | 198 | 57 |
| WL5422 | ACCGAATCCAAGCTGATCCT | CTCCTGTTCTGAGTTGGTGC | 1J2 | 204 | 57 |
| WL4908 | TTTGCCTCCCAACCATTTAC | ACAACCTCTGCTTCCCTCTG | 1 | 218 | 57 |
| WL5430 | TGACAGTGGCGGATCTAGAA | AAGACCCTATCGTGTGGCAG | 1J1 | 207 | 57 |
| WL5414 | ACCGGTTCACCCTTGGAAAA | TGGACTGAGAGGAAGTTCGG | 1J2 | 194 | 57 |
| WL5416 | TCACCTGTTCACCCTTGGAA | TTTGCAGACGATGATTGGGG | 1J1 | 157 | 57 |
| WL5412 | GCCTCTAGAAACTCCCCTCC | GTTTTGAATCGGGCAGCGAT | 1J1 | 229 | 57 |
| WL5049 | CCCACCCGTATCCATCCTC | GCCGAAGAGCAGGAAGGT | 1J2 | 177 | 57 |
| WL5410 | GCGTTTATTGAGCTTCCGGT | TTTTCCTCCGTTCCAGTGTT | 1J1 | 650 | 57 |
| WL5406 | TGCGCCCAGTGTATATAGCA | TGCATCTCCCACACTCATCA | 1J2 | 237 | 57 |
| WL4966 | TTTATGCCCAATCTTGTGTTCAT | CCAAAGCCCTTCCTGCAA | 1J2 | 206 | 57 |
|  |  |  |  |  |  |
| WL4880 | TAGCACACCTACCACGGACA | GGCAAATAAGGCTGAGGTGA | 2J1 | 166 | 54 |
| WL5607 | CCTTCGAGTGCCACTAACCT | CAAGAGCAAGTGACTAGCGC | 2J1 | 243 | 57 |
| WL5609 | TGGAACCGGATGATTTGGGG | GGACTATTGGATTGCGACTCG | 2J1 | 113 | 57 |
| WL5611 | GCAACCAGTGAGATCGTTCA | AACGCCAGACCCCAAATCAT | 2J1 | 143 | 57 |
| WL4593 | TTAATGCTGGAAGACGCC | TTTCCTTTGCGGATAGCAGATAG | 2J2 | 330 | 55 |
| WL4591 | CTTGCCCCGTCGATTTAC | ATTGTTAATGGTGGATGAGAAAG | 2J2 | 306 | 55 |
| WL5613 | CAGAACGCTCACAGTGACC | ACTTGACTGGCCCTGTATTG | 2 | 106 | 57 |
| WL4938 | CATTGCCATTCACAGCATTT | TAGGTTTCGCGGTGAGAATA | 2J2 | 246 | 54 |
| WL5615 | CTAAGTTTGTGCGTTGCCTG | CGTGTATCTGTCGTGAGTGG | 2J1 | 298 | 57 |
| WL5091 | CATCTCATTATCACCACTGTTCG | GGGCCTGATGCTAATCTCTT | 2J1 | 527 | 57 |
| WL4665 | CTTGAAACGGCCCCTACTTTTT | GCTTTTCATGTGCCTAGTTATTG | 2J1 | 600 | 55 |
| WL4619 | GTTATGGGTGTCAGTGAGGTG | GTGAACATCGCTGGAATTGG | 2J2 | 290 | 55 |
| WL5400 | CACAGGCAGACTCGCAATT | AGTCACACAGCTCAGTACCC | 2J2 | 220 | 57 |
| WL5396 | CCGAATCTGCTGCGAAATAATT | ACCCTTGTCCGTTTAGTTCAC | 2J1 | 130 | 57 |
| WL5617 | GCGGTCAATGGCTTTAATTTCC | GCCAAACCTTTTCCTGCCTA | 2J2 | 291 | 57 |
| WL5619 | CGGGTAAACTCGCTAATGAAACT | TGTGATGTGTCTGTGTTGTGT | 2J2 | 156 | 57 |
| WL5031 | AAGTCCCCATTCCTCAGCAT | TCGCCATCAAAACCCTCTAC | 2J1, 4J2 | 166 | 59 |
| WL4968 | CGCGAATCGTCTTGCTGAAA | AGTCGAAGTTGCATTCCAGTG | 2J1, 4J2 | 166 | 57 |
| WL5051 | AGACGTTCAACCTCGATAGCA | TGTGAATTGCAGGTAGGGGA | 2 | 248 | 59 |
|  |  |  |  |  |  |
| WL5627 | GGACGACAGTTTACCAGACATC | ACATCTACCCAACTGCCCG | 3J1 | 503 | 57 |
| WL5623 | GCTTTCGGTCAATAAATCTAGCG | TCAAACTAAAAGTCCGCAACAA | 3J1 | 176 | 57 |
| WL5129 | TCGAGCGAGCCTTACGATTC | GGGGCTGCAAACGTTCTTAA | 3J2 | 234 | 57 |
| WL5079 | TGTAAACACCTAACAACCGCT | AATGCGCCATACAACTCAGG | 3 | 340 | 57 |
| WL5631 | TTCCGTTCTCTGACACTCCC | AGAGAGAGAGAGGGAGGTGG | 3J1 | 105 | 57 |
| WL4832 | GGGACGGTACTCCCTCTTCT | TCCAACGGATAGTTCCTGGT | 3 | 153 | 54 |
| WL4940 | AGAGTTTGGGGCTGAATCCT | TCCACCAAGGTCAGAACACA | 3J2 | 218 | 54 |
| WL5093 | GTCGGTTCCTTGATTGCGTC | AGTAAGATTTGACTGCGTGCA | 3J1 | 218 | 57 |
| WL5081 | TGAACTTGAAGTGGATGCTCA | AGGAACGAGACAACATCACATAT | 3J2 | 181 | 57 |
| WL5135 | ACCCGTAACGAACACTTGATG | CTCACCCGTTTCTGCGTC | 3J1 | 248 | 57 |
| WL5392 | ACGGGACACTCAATAGAACTGT | GTAAGCCCTAACCCGTTGC | 3J2 | 283 | 57 |
| WL5133 | CGATACATAGCACCCTCCGT | CTTTGCTTAGCCAGGTCCAC | 3J2 | 169 | 57 |
| WL5388 | GGAACACCCTTTCTTCAGTCC | TAGCTATGTGGGCCAAGGAA | 3J2 | 159 | 57 |
| WL5390 | AGAAGATCGACCGCCACAA | GTCGCTGTTGTGCTACTCG | 3J2 | 203 | 57 |
| WL4513 | GCTGTTTGCATTCACTTGCT | TTGTGTGAGTTTATGTGTGTGTG | 3J1 | 449 | 53 |
| WL4511 | TGCCTACACAAAGATGGAAGC | GCTCTGCAATTCTGCTTGTT | 3J1 | 373 | 53 |
| WL5057 | AGTCCTCTTCCTCATGCCAG | GTCCGCCTAGCCTTCAGTT | 3J1, 3J2 | 371 | 57 |
| WL5384 | TGGTTAGGGTCGTACTCACTG | CGACACGCACGTTATAGAGA | 3J1, 3J2 | 177 | 57 |
|  |  |  |  |  |  |
| WL5123 | GTCCGCCACAACGTATGA | TCGAGACAACATTCAGCGAA | 4J1, 4J1 | 358 | 57 |
| WL4539 | ATCCAGGGGGTAAGCAACA | GTGCTGAATCGGTGTGGTTT | 4J2 | 443 | 54 |
| WL4515 | AAGTTCTAGGGGCAAAAAGGA | CTACGGAGATGCCGATCAA | 4J1 | 310 | 53 |
| WL4663 | AATGCCTATGAAGACAGGGTAAA | GTTGTGTGGCAGTTGTCGTATAG | 4J2 | 316 | 55 |
| WL4661 | ATGCTGTGATCGGTTCGGT | TTTTTGGCACGAAACAAACT | 4J1, 4J2 | 530 | 55 |
| WL4914 | TCTTATCCGCACGTTACAGC | GATTTCCCAGGATGCAACAC | 4J1 | 210 | 54 |
| WL5033 | AATCTCATGTGCGTGCAATG | CACAAAGCTTAACGTGTACTGT | 4J2 | 442 | 57 |
| WL5125 | AGCAGAAAGAGAGAGGTGAAGT | GAGCATCCTAGTCAAAGTAACCA | 4J1 | 286 | 57 |
| WL4523 | GCAGAACCATCGCCATCTC | GGGAAGCGGACTCTAAAAGAA | 4J2 | 355 | 53 |
| WL5160 | GGCAATCGGAGCATACACAT | ATACGGGGAAATGCGAGGTT | 4 | 248 | 59 |
| WL5059 | GGTAAGTTGGTAACCCTGTAAGT | CTCGTTCAAGGCCAGTATTTGA | 4J1 | 474 | 57 |
| WL5035 | GGAAAGGCGTCGATGGATAC | GGTAGAGCTGTAGACCGTCG | 4J1, 4J2 | 201 | 57 |
| WL4972 | ACCATGAATCGGGCACAGTA | AGATCAGATTACACACCTGCAA | 4J1, 4J2 | 299 | 57 |
| WL5053** | CTCAGCGAGGATCAGACAATGC | AATGGCCAGAGAAACGAGAAAGAG | 4 | 145 | 53 |
| WL5055** | TGTATGTATGTTTGTTTCGTCCTTTG | CTGTGAGCACACATCACGAGTAAG | 4 | 82 | 53 |
|  |  |  |  |  |  |
| WL4517 | ATCCAACTAATTTTGTAAGCGTTAGC | CTACGAGAGCAAGCAGGAGG | 5J1 | 167 | 53 |
| WL5633 | GGACCTGCCCTGCCTAAAT | TGGAGAGTTGACCGGTTGAT | 5 | 224 | 57 |
| WL5440 | TAATGTTGGCTTGCATCCGG | ATGACCTCGGCTATGACTGG | 5J1 | 245 | 57 |
| WL5679 | CCCTCTAGGCTTCTGTGGG | GCTGAGATGAAAGTCGGACA | 5J1 | 943 | 57 |
| WL5635 | GTTCAGTCATGGCCTTCTCG | ATGCAAACAACTACCCCAGC | 5J1 | 123 | 57 |
| WL5637 | AAAATCTGTGCCCGACGTAT | AGAAGGGTGGTTGCTGACTA | 5 | 154 | 57 |
| WL5675 | GGGTTGCTCATCTTCCGAATAC | TGTCCTACTTGTTCTGCACA | 5J1 | 190 | 57 |
| WL5673 | GGAGACAGGCTGGACAGAAA | TTTTACCAAGCACGAACGTT | 5J1 | 170 | 57 |
| WL4625 | TATGGCTTCATTACCTGTGTTG | CTTTGTTCCCATCATTCTCCTTG | 5J2 | 639 | 55 |
| WL4627 | ACTTGCTGGAAAAACAAGCGATA | GGGACTTTGTTTAGATTTCCAAC | 5J2 | 216 | 55 |
| WL5651 | GGCCCTCAAAGATATAACGGTTT | AGTCGCGGCCTCATAGTTTT | 5 | 136 | 57 |
| WL4930 | CAACGGATTTCATCGAGTGT | GTGGTGTTCTTGCTCCCTCT | 5 | 212 | 57 |
| WL5172 | ATAACGCTAGAGACTCCGCC | GAGATCTACGCCACGACTGA | 5J1 | 196 | 57 |
| WL5170 | CCCTTCTTCGACCTACAGCT | GTGATCGACGGGGCTAGAG | 5J1 | 248 | 57 |
| WL5438 | ACCGTCTCACAGCATATGGT | TCGATACCCAGCATCTCCAT | 5J1 | 157 | 57 |
| WL5677 | CGGCTATCTAACCATGAATCCC | CTCTTGCTTCTACGGACTGA | 5J2 | 122 | 57 |
| WL5137 | TTGTGGAATGATCAGTAAACGC | ACACACACCCCAATTCCTGT | 5J1, 5J1 | 500 | 57 |
| WL5436 | TGGGAATTAATGTGGGGCCT | CGCTCTAAATTTCCCACGGG | 5J2 | 166 | 57 |
| WL5083 | GACGGAGAGCGGAGTGTACC | TTTTGCTCCATACCATCCACCT | 5J1 | 163 | 57 |
| WL5085 | TGGAATTACAAATGGATCGATGG | TCATTCAGAAAGCTTGGACAATA | 5J1 | 270 | 57 |
| WL4974 | ACAGCTTGGGTCCATTCTCT | GCTAGTCCACTTAATTCCACCA | 5J1 | 249 | 57 |
| WL4537 | AGATTTGCACTAACACGGGAA | TCGTTCCCCTCGCTTGTAGT | 5J1 | 400 | 54 |
| WL4830 | TACCATCCAGCCTAACCGAC | CTGGCCCTCTCTATGCACAT | 5J2 | 717 | 54 |
| WL5009 | GTCGATGTTCAGCTGGCA | GACGAGGGTGGGGAAGTG | 5 | 700 | 57 |
|  |  |  |  |  |  |
| WL4902 | AGACCTTGCCGGAATCAAC | CAACCCACATTGCCTTCTCT | 6J1 | 201 | 54 |
| WL4904 | TCTGGAGGCCCACTATTGC | GGTTCGCCAGTTTGCTCTT | 6J1 | 246 | 54 |
| WL4918 | ATCACGACTGAGAGGGGAAA | TTGGTGTGAGCTTGGTTTGA | 6J1 | 215 | 57 |
| WL5643 | TCTCTCCCTCTCTCTCTTAGA | GGCAACGCACCAAGATAATC | 6J2 | 133 | 57 |
| WL5312 | CTGCTTGGTCGCTGTATGTC | CTCGATCAACCGCTTACCAC | 6 | 202 | 57 |
| WL5314 | TGTCAGTTGGCCCATCAGAA | GTACTGGCCTATCGGAGCAG | 6 | 169 | 53 |
| WL5497* | GAAGACTGGTAGCGATGCAGTCA | AGAACAGATACACCTCCACTGACCA | 6J2 | 362 | 52 |
| WL5499* | TCAGTTGATGTTACTGAAGATCTGAAGT | TTCGGATACATCAGATGGACCAT | 6J2 | 119 | 52 |
| WL5501* | CCAGGAGGGCCTCCAGGA | CTGGGAGCTCCTGCTGCGT | 6J2 | 390 | 55 |
| WL5503* | CCAACTCTAGCTGACCGCAGACTA | ATTCCATGGTGTAATAGCTCCAACTAC | 6J2 | 459 | 52 |
| WL5316 | CAGAGCGACAGATTCAACGG | CTGGAGCCATTGAAGCAGTG | 6 | 239 | 57 |
| WL5067 | CCCTACACGCCTGTTTTGTC | TGCAGAGTCACAGCCTTACA | 6J1, 6J2 | 163 | 57 |
| WL5039 | CTACTACGAGCGATTGGTGC | CTACTTTATTACCCGGCGCG | 6J1 | 240 | 57 |
| WL4976 | GACTCGTACAAACACCATCTGA | CTGTTCCTCACACCCCTCTA | 6J1 | 880 | 57 |
| WL4531 | ATATGGAAGAATAACGAACAGCA | GGTTCGGATTGCAGGGTT | 6J2 | 680 | 54 |
| WL4533 | ATATGGAAGAATAACGAACAGCA | GGGCCTTTATGAATTGGCT | 6J2 | 267 | 56 |
|  |  |  |  |  |  |
| WL4503 | TACAACAGCAACAGAAGTGT | AGACACAGCTCCTCATCAACG | 7J2 | 386 | 55 |
| WL5337 | GATCAGCCCTATCCAAGCAA | AGGTACAAGCAGCCTTAGTGT | 7J2 | 180 | 57 |
| WL5341 | GCCGCTAACCATCCTCAACT | GCCTAGATGACCAGCCCTTA | 7J2 | 152 | 57 |
| WL5356 | TAAGGTCAGGTTGGCTTCGG | TTCGTCGCGGCCCTAAAA | 7J2 | 242 | 57 |
| WL5358 | GCAATCAGGTGTGGGTTTCC | AACTGAGCGAGCTGGATGAT | 7J2 | 250 | 57 |
| WL4934 | TATCCCATTGTCACCACGAA | AGGCTGAGGGTTTGTACCAG | 7 | 247 | 57 |
| WL5041 | GAATAGGGTTGGCGTCGTTC | TCTGGGTCTGAACTTGCACT | 7J1, 7J2 | 214 | 57 |
| WL4503 | TACAACAGCAACAGAAGTGT | AGACACAGCTCCTCATCAACG | 7 | 450 | 53 |
| WL4505 | GTAAGCTGCTCTCACCTGCAT | GGCCCATAAACTGGAACCCT | 7J1 | 487 | 57 |
| WL5174 | GTGCAAGCGTCGGTGAATT | TGGTTTATTCGTTTTCAGCCGT | 7J2 | 215 | 57 |
| WL5428 | CGCCATGTCTACGAAAGCAT | GCAGGCACACATACGATCTC | 7J1 | 240 | 57 |
| WL4623 | GCGAATAACGGACATCACTAGC | GATCGAGGTCATGGTACACTTGG | 7J2 | 475 | 55 |
| WL5073 | GCTCATTCGCAAATTCACCG | GCAGACCGGCCAAATCAA | 7J2 | 564 | 57 |
| WL5645 | GACACGAAGGATTGAACCCG | GGGATGAATACGCGGAGACT | 7J2 | 300 | 57 |
| WL5647 | TGGACAGATAGCCCGATCAC | CAGGGCAGTCGATTTGATGG | 7 | 211 | 57 |
| WL5071 | CGTTTATGTGCTGGATCGGG | GGCCCAGCTTGTCATTTTGA | 7J1, 7J2 | 197 | 57 |

* Adopted from (He *et al.*, 2013)

** Adopted from (Kaur *et al.*, 2008)

**Table S2.** Distribution of the number of markers specific to each subset of the species evaluated

| Species positive for markers | Number of markers |
| --- | --- |
| SWG | 2 |
| SWG, TB | 14 |
| SWG, DV | 6 |
| SWG, TE | 3 |
| SWG, TB, TE | 3 |
| SWG, TB, DV | 1 |
| SWG, TE, DV | 1 |
| SWG, TB, TE, DV | 2 |

SWG- Sea wheatgrass, TB- *Th. bessarabicum*, TE- *Th. elongatum* and DV- *D. villosum.*

| **Table S3**. List of markers used in the three rounds of screening | | |
| --- | --- | --- |
| First Screen | Second screen | Third screen |
| WL4569 | WL4966 | WL5087 |
| WL4591 | WL4581 | WL5063 |
| WL4511 | WL4938 | WL5166 |
| WL4523 | WL4880 | WL4583 |
| WL4515 | WL4665 | WL5079 |
| WL4533 | WL5057 | WL5093 |
| WL4505 | WL4940 | WL5135 |
|  | WL4663 | WL5129 |
|  | WL4972 | WL5033 |
|  | WL4914 | WL5123 |
|  | WL4830 | WL5133 |
|  | WL4974 | WL5059 |
|  | WL4625 | WL5035 |
|  | WL5009 | WL5170 |
|  | WL4904 | WL5067 |
|  | WL4918 | WL5039 |
|  | WL4623 | WL4976 |
|  | WL5043 | WL5041 |
|  |  | WL5071 |
|  |  | WL5091 |
|  |  | WL5174 |
|  |  | WL5073 |

**Table S4.** Numbers of markers specific to individual SWG chromosome

| Homoeologous groups | J_1_ genome-specific markers | J_2_ genome-specific markers | J_1_ and J_2_ markers | Total |
| --- | --- | --- | --- | --- |
| 1 | 6 | 10 | 2 | 18 |
| 2 | 8 | 7 | 2 | 17 |
| 3 | 8 | 5 | 3 | 16 |
| 4 | 4 | 4 | 4 | 12 |
| 5 | 11 | 6 | 1 | 19 |
| 6 | 5 | 7 | 1 | 13 |
| 7 | 3 | 9 | 1 | 14 |
| Total | 45 | 48 | 14 | 107 |
